# What is the safe and effective dilator number during access in PCNL? Three-shot dilation versus classical sequential Amplatz dilation

**DOI:** 10.1186/s12894-023-01368-6

**Published:** 2023-11-29

**Authors:** Omur Memik, Bekir Voyvoda, Murat Ustuner, Onur Karsli, Ahmed Omer Halat, Levent Ozcan

**Affiliations:** 1 Department of Urology, University of Health Sciences, Kocaeli Derince Training and Research Hospital, Lojman Sokak, Derince, Kocaeli 41900 Turkey; 2 Department of Urology, University of Health Sciences, Prof. Dr. Cemil Tascioglu City Hospital, Istanbul, Turkey

**Keywords:** Percutaneous nephrolithotomy, Tract dilation, Fluoroscopy time, and nephrolithiasis

## Abstract

**Background:**

Although PCNL has been used for a long time to treat nephrolithiasis, there is still contradictory information concerning the use of the dilation method. In this study, we aimed to compare conventional sequential Amplatz dilatation (SAD) using ten dilators and a method using three dilators (12, 20, and 30 Fr), which we named “three-shot dilatation” (3SD), in terms of fluoroscopy time (FT), operation time, bleeding and stone-free rates.

**Methods:**

The study included patients who underwent PCNL with the SAD and 3SD methods. A different surgeon with extensive endourology experience applied each technique. One of the surgeons operated on the patients using the SAD method with ten dilators, and the other surgeon performed the operations using the 3SD method involving three Amplatz dilators (12, 20, and 30 Fr).

**Results:**

A total of 283 patients, 138 in the 3SD group and 145 in the SAD group, were included in the study. The mean age of the patients was 47.32 ± 13.71 years. There was no statistically significant difference between the two groups regarding preoperative characteristics (*p* > 0.05). The FTs of access 2, total access, and total operation were significantly shorter in the 3SD group (*p* = 0.0001). The decrease in hemoglobin was statistically significant in the 3SD group compared to the SAD group (*p* = 0.022), while the blood transfusion requirements of the groups were similar (*p* = 0.176). There was no statistically significant difference between the two groups regarding stone-free rates (*p* = 0.973). In four patients in the SAD group, re-access was necessary due to the loss of passage due to the guide wire slipping out of its place.

**Conclusion:**

Intraoperative FT can be shortened using the described 3SD method without compromising surgical safety. However, this method can be used as an intermediate step in the transition to one-shot dilation by surgeons experienced in performing SAD.

## Background

Due to the high success rate, short hospital stay, rapid postoperative recovery, and minimal renal parenchymal damage, percutaneous nephrolithotomy (PCNL) has become one of the most preferred minimally invasive methods. It has replaced open surgery in treating kidney stones larger than 2 cm [1, 2]. The most common complications associated with PCNL, such as renal bleeding, insufficient tract dilatation, and collecting system perforation, occur during nephrostomy tract creation. Therefore, creating the nephrostomy tract constitutes the crucial step of PCNL operations [3]. In the literature, various methods have been described for dilation, including sequential Amplatz dilatation (SAD) [4], metal telescopic dilatation (MTD) [5], and balloon dilatation (BD) [6]. In addition, Frattini et al. defined the “one-shot dilatation” (OSD) method, which, similar to BD, provides tract creation in a single step using a 25 or 30 F Amplatz dilator to reduce X-ray exposure during fluoroscopy use in PCNL [7].

MTD and SAD are sequential dilatation methods in which dilators of increasing thickness are first inserted into and then withdrawn from the renal parenchyma through the collecting system. However, it has been suggested that the use of MTD and SAD for dilation purposes prolongs tract dilatation, thus increasing the radiation exposure time and tract loss during dilatation, and that the perforation of the collecting system and the loss of the tamponade effect on the renal parenchyma tract while changing dilators may cause more blood loss [8, 9]. In addition, using both metallic and sequential fascial dilators increases the dilatation time and the incidence of guidewire bending during tract creation, which may prevent adequate dilation [10]. Balloon dilators have the advantages of easy use and fast application. They can expand up to a predetermined diameter in the radial plane in a single attempt without repetitive entries in the longitudinal axis, thus reducing the risk of parenchymal damage and bleeding. They also create a fast access line in a single step, minimizing other risks, such as guide wire breakage and the loss of access line, which can be observed in repetitive procedures [11]. However, this method also has certain disadvantages, including a higher cost and the need to be reusable [12]. In the literature, some studies have shown that the OSD method is as safe and effective as standard techniques and takes less time, while others suggest that it may cause more parenchymal damage than gradual dilation [8, 13]. Although PCNL has been used for a long time to treat nephrolithiasis, there are still contradictory data concerning the best dilation method to use during PCNL [14, 15]. The surgeon’s experience, patient-related factors, and possible complication risks affect selecting these methods.

Nevertheless, when costs are considered, sequential dilatation with Amplatz dilators remains one of the most preferred methods [3]. A standard Amplatz dilatation set consists of 10 dilators, ranging from 10 to 30 Fr, and three sheaths. However, studies comparing SAD and OSD have not specified the exact number of dilators used in SAD or whether they used lapsed dilation. Therefore, it has yet to be clarified how many dilators should be used during SAD and what the standard should be.

This study aimed to investigate the efficacy and safety of the three-shot dilatation (3SD) method using 12, 20, and 30 Fr Amplatz dilators as compared to the conventional sequential dilatation method using 10 Amplatz dilators (12–30 Fr) in PCNL.

## Method

Five hundred sixty-five patients were treated with PCNL from 2012 to 2022 in our center. Of these patients, 283 whose operation and fluoroscopy times (FTs) were documented in detail were included in the study. Exclusion criteria from the study were patients with renal and skeletal anomalies, solitary kidneys, multi-tract dilation, missing FTs (cases in which only total access and total operation FTs were available), and bleeding disorders. All patients were evaluated based on a detailed medical history, physical examination, complete blood count, serum biochemistry, urine analysis, urine culture, and coagulation tests. Each patient also underwent a comprehensive preoperative and postoperative radiologic assessment, including plain abdominal radiography and non-contrast and contrast-enhanced computed tomography (CT). Each of the two methods was applied by a different surgeon with substantial experience in the field of endourology who received urology residency training in different clinics and was accustomed to using a different number of dilators during access dilation. Both surgeons operated on patients with PCNL indications who presented to their outpatient clinics by making appointments through the central appointment system. One of the surgeons used the SAD method, and the other used the 3SD method while performing PCNL.

Since the definition of FT during access dilation differs in the literature, we made our definition. We recorded four FTs in total: access 1 FT (insertion of the needle to the placement of the guidewire into calyx), dilatation/access 2 FT (from the advancement of the style over the guide wire to the placement of the Amplatz sheath), post-access FT (from the start of nephroscopy to nephrostomy catheter placement), and entire operation FT (from the insertion of the needle to nephrostomy catheter placement). The patient’s demographic data, stone sizes, access times (from the insertion of the needle to the placement of the Amplatz sheath), total operation times (from the insertion of the needle to nephrostomy catheter placement), four FTs described above, grade of hydronephrosis, estimated blood loss, stone-free rates, number of blood transfusions, intraoperative and postoperative complication rates, and nephrostomy catheter removal and discharge times were statistically compared between the SAD and 3SD groups. Hydronephrosis was graded as either absent, mild, moderate, or severe based on the renal ultrasound scan and IVU [16]. For each patient, measured hemoglobin concentrations before the surgical procedure and 4 and 24 h postoperatively were recorded. The estimated stone size was calculated using Ackermann’s formula, volume = 0.6 x π x r2, where r is half of the largest diameter of the stone. All patients were re-evaluated with non-contrast CT, routinely taken the first month postoperatively. The success of PCNL was defined as stone-free status or the presence of residual stone fragments smaller than 4 mm [17]. Complications were graded according to the Clavien-Dindo classification system, including five grades [18].

### Surgical technique

In all patients, an open-ended 5 F ureteral catheter (MarflowTM, Marflow AG, Switzerland) was inserted in the lithotomy position as a standard. The anatomy of the pelvicalyceal system was visualized with radio-opaque material that was instilled using the ureteral catheter under C-arm fluoroscopy. A 19.5-gauge percutaneous needle (Percutaneous Access Needle, Boston Scientific Corporation, MA, USA) was introduced in the prone position, and Amplatz (Boston Scientific Microvasive Amplatz TractmasterTM, Boston Scientific Corporation, MA, USA) dilatation was started over the guide wire. Dilatation was performed using 12, 20, and 30 Fr dilators sequentially in the 3SD group and the even sizes of 12 Fr to 30 Fr dilators sequentially in the SAD group. The Amplatz sheath was placed, and the nephrostomy tract was entered using a 28-F rigid nephroscope (Karl Storz™ Endoscopy-America Inc.). The stones were fragmented with a pneumatic lithotripter (Calculith™ Lithotripter, PCK, Turkey) and removed using forceps. The operation was completed by placing a 14-Fr nephrostomy catheter in the kidney. The ureteral catheter was extracted, and the nephrostomy tube was clamped on the first day following the operation. Without of fever or urinary leakage from the nephrostomy tract, the nephrostomy tube was usually removed on the second postoperative day, and the patient was discharged the following day.

### Statement of ethics

This study was approved by the Institutional Review Board of the Health Sciences University Kocaeli Derince Training and Research Hospital. Written informed consent was obtained from all patients. All procedures related to humans complied with all the relevant national regulations, institutional policies, and tenets of the Declaration of Helsinki.

### Statistical analysis

The Statistical Package for the Social Sciences (SPSS) for Windows v. 20.0 (SPSS Inc., Chicago, IL) was used for statistical analyses. The independent-samples t-test (for parametric data) and the Mann–Whitney U test (for non-parametric data) were used to compare measurable values between the groups. The chi-square test was used to compare categorical values between the groups. Statistical significance was accepted at *p* < 0.05.

## Results

A total of 283 patients, 138 in the 3SD group and 145 in the SAD group, met the study’s inclusion criteria. There was no statistically significant difference between the groups regarding preoperative characteristics (*p* > 0.05). The preoperative data and statistical analysis of the study groups are shown in Table 1.

**Table 1:** Demographic data and stone parameters of the study groups

	Demographic variables		
	3SD (*n* = 138)	SAD (*n* = 145)	*p*
Age (mean ± SD)	47.48 ± 13.25	47.16 ± 14.18	0.844^1^
BMI (kg/m^2^) (mean ± SD)	24.88 ± 2.97	24.47 ± 2.5	0.206^1^
Gender (n)			0.606^2^
* Male*	101 (73.2%)	110 (75.9%)	
* Female*	37 (26.8%)	35 (24.1%)	
Laterality (n)			0.848^2^
* Right*	66 (47.83%)	71 (48.97%)	
* Left*	72 (52.17%)	74 (51.03%)	
History of surgery (n)			0.609^2^
* Absent*	116 (84.06%)	127 (87.59%)	
* Open*	8 (5.8%)	8 (5.52%)	
* Endoscopic*	14 (10.14%)	10 (6.9%)	
ESWL (n)	44 (31.88%)	36 (24.83%)	0.188^2^
Stone location (n)			0.430^2^
* One calix*	21 (15.22%)	19 (13.1%)	
* Pelvic*	37 (26.81%)	53 (36.55%)	
* Multiple calyces*	5 (3.62%)	7 (4.83%)	
* Pelvic and calix*	61 (44.2%)	52 (35.86%)	
* Staghorn*	14 (10.14%)	14 (9.66%)	
Hydronephrosis grade (number)			0.571^2^
* No hydronephrosis*	24 (17.39%)	22 (15.17%)	
* Mild*	71 (51.45%)	83 (57.24%)	
* Moderate*	34 (24.64%)	35 (24.14%)	
* Severe*	9 (6.52%)	5 (3.45%)	
Stone burden, mm^2^ (mean ± SD) (median)	603.7 ± 543.55 (423)	578.99 ± 554.13 (423)	0.399^3^
Stone density in Hounsfield units (mean ± SD) (median)	1175.96 ± 363.94 (1171)	1216.83 ± 377.61 (1200)	0.506^3^

*3SD* Three-shot dilation, *SAD *Sequential Amplatz dilatation, *SD *Standard deviation, *BMI *Body mass index, *ESWL *Extracorporeal shock wave lithotripsy

^1^The Independent Samples t Test

^2^Chi-square test, ^3^Mann-Whitney U test

Access 2 FT, total access FT, and total operation FT values were significantly shorter in 3SD than in the SAD group (*p* < 0.05). However, the two groups had no significant difference regarding access 1 FT and post-access FT (*p* > 0.05). The data of FTs according to the study groups are detailed in Table 2.

**Table 2:** Distribution of FTs according to the study group

	3SD (*n* = 138)	SAD (*n* = 145)	p*
Access 1 FT^a^ (mean ± SD) (median)	9.22 ± 5.88 (8)	9.41 ± 5.07 (8)	0.275
Access 2 FT^b^ (sec) (mean ± SD) (median)	45.99 ± 22.16 (41)	71.86 ± 20.79 (69)	0.0001**
Total access FT (sec) (mean ± SD) (median)	55.21 ± 23.42 (50)	81.26 ± 21.56 (80)	0.0001**
Post-access FT^c^ (sec) (mean ± SD) (median)	17.09 ± 15.16 (13)	20.59 ± 30 (11)	0.054
Total operation FT (sec) (mean ± SD) (median)	72.36 ± 28.58 (66)	101.83 ± 39.96 (90)	0.00011**

*FT* Fluoroscopy time, *3SD *Three-shot dilation, *SAD*: sequential Amplatz dilatation, *SD* Standard deviation

*Mann-Whitney U test

**statistically significant

^a^FT from the insertion of the needle to the placement of the guidewire into the calix

^b^FT from the advancement of the style over the guide wire to the placement of the Amplatz sheath

^c^from the start of nephroscopy to nephrostomy catheter placement

Total access time was significantly shorter in 3SD than in the SAD group (*p* < 0.05). However, the two groups had no significant difference regarding total operation time (*p* > 0.05). Table 3 presents detailed data on the groups’ total access and operation times.

**Table 3:** Total access and total operation times of the study groups

	3SD (*n* = 138)	SAD (*n* = 145)	*p*
Total access time (sec) (mean ± SD) (median)	266.01 ± 50.17 (263)	443.42 ± 54.92 (440)	0.0001^1^*
Total operation time (min) (mean ± SD) (median)	54.71 ± 37.63 (43)	60.1 ± 42.47 (45)	0.334^1^

*3SD* Three-shot dilation, *SAD *Sequential Amplatz dilatation, *SD *Standard deviation

^1^Mann-Whitney U test

*statistically significant

When grade 1 complications were examined, postoperative fever was observed in six patients in the 3SD group and five in the SAD group. Concerning grade 2 complications, urinary tract infections were observed in three patients in the 3SD group and four in the SAD group, and all these patients were treated with appropriate antibiotic therapy. Six patients in the 3SD group and 12 in the SAD group required blood transfusions. When grade 3 complications were examined, ureterorenoscopy was required in four patients, each in the 3SD and SAD groups, due to the stones migrating to the ureter. After removing the nephrostomy catheter, double-J stent insertion was required in two patients in the 3SD group and three in the SAD group due to prolonged urinary leakage (leak of urine from the nephrostomy tract for more than 24 h after nephrostomy tube removal). Due to uncontrollable bleeding, selective angioembolization was performed on one patient in each group. No Clavien grade 4 or 5 complications were observed in patients (*p* = 0.585, Table 4). In four patients in the SAD group, re-access was necessary due to the loss of passage due to the guide wire slipping out of its place. The postoperative data and statistical analysis of the study groups are shown in Table 4.

**Table 4:** Postoperative parameters of the study groups

	3SD (*n* = 138)	SAD (*n* = 145)	*p*
Hospital stay (day), mean ± SD (median)	3.44 ± 1.44 (3)	3.89 ± 1.49 (4)	0.004^1^*
Time to nephrostomy catheter removal (day), mean ± SD (median)	2.43 ± 1.20 (2)	2.88 ± 0.86 (3)	0.0001^1^*
Urinary leakage after nephrostomy catheter removal (hour),	6.7 ± 10.30 (4)	7.08 ± 10.30 (5)	0.025^1^*
Hemoglobin drop (g/dl), mean ± SD (median)	1.27 ± 0.89 (1.1)	1.63 ± 1.28 (1.3)	0.022^1^*
Blood transfusion, n (%)	6 (4.35%)	12 (8.28%)	0.176^2^
Complications, n (%)			0.585^2^
* Clavien grade 1*	6 (4.35%)	5 (3.45%)	
* Clavien grade 2*	9 (6.52%)	16 (11.03%)	
* Clavien grade 3*	7 (5.07%)	8 (5.52%)	
Stone-free status, n (%)	122 (88.41%)	128 (88.30%)	0.973^2^

*3SD* Three-shot dilation, *SAD *Sequential Amplatz dilatation, *SD *Standard deviation

^1^Mann-Whitney U test

^2^Chi-square test

*statistically significant

## Discussion

Although PCNL is a minimally invasive surgical technique, exposure to X-rays remains problematic for surgeons, patients, and operating room personnel. In many centers, access is still provided under fluoroscopy and its alternatives, such as ultrasonography and CT. This has made radiation exposure an essential issue during PCNL [19–21]. In a study, Dong et al. reported effective and safe surgery without radiation exposure using an ultrasound-guided two-step dilatation technique [22]. In another study, two-step dilatation was proposed by Xu et al. to minimize the risk of access failure and to prevent adverse complications such as renal pelvic perforation, extravasation, and bleeding, especially in patients with an undilated collecting system [23].

Since kidney stone disease is recurrent, and patients may require more than one procedure during their lifetimes, it is crucial to reduce their exposure to X-rays [24]. It has been reported that shortening access time through single-step dilation (OSD or BD) during tract creation decreases X-ray exposure, thus reducing operation time [10, 24, 25]. In light of this information, although single-step or tract dilatation involving a lower number of dilators reduces the FT, most studies focus on comparing OSD and SAD. However, there needs to be more precise information in the literature concerning whether all or how many dilators were used in patients undergoing SAD during PCNL. Although there are studies suggesting that OSD is effective and reliable, it may not be realistic to expect a surgeon who routinely performs PCNL with SAD to change his or her accustomed technique and perform dilation with a single 30-Fr dilator (OSD) rather than starting with a 10 F dilator and using ten dilators (SAD) in order to reduce the FT.

Total FT is a poor indicator of dilatation FT since the duration of fluoroscopy during the placement of the guide wire by entering the collecting system through a needle, and the control of residual stones may differ in each case [7]. However, a recent meta-analysis comparing four different dilatation methods stated that the heterogeneity between dilatation FTs was due to the different definitions of the duration of dilatation fluoroscopy in studies [12]. Therefore, the most accurate definition for the duration of dilatation fluoroscopy is the time from the advancement of the style over the guide wire to the placement of the working sheath. In the current study, we defined the stages of fluoroscopy during the formation of the nephrostomy tract as access 1 FT and access 2 FT. We recorded the FTs at these stages separately. To the best of our knowledge, this study is the first to compare differences in the use of different numbers of dilators for dilatation in patients undergoing SAD during PCNL.

When we compared both groups, the access 2 FT, total access FT, and total operation FT values were significantly shorter in 3SD than in the SAD group. However, the two groups had no significant difference regarding access 1 FT and post-access FT. Although the total operation time was shorter for the patients in the 3SD group than those in the SAD group, there was no statistically significant difference.

With the widespread use of PCNL in treating kidney stones, bleeding has become one of the most common and worrisome complications [26]. A correct puncture route and a proper tract dilatation method are the main factors determining the amount of intraoperative blood loss [27].

Miniaturization in PCNL was inspired by attempts to reduce blood loss during PCNL by reducing the size of the tract and, consequently, parenchymal and infundibular trauma. Miniperc was defined by Jackman et al. as a percutaneous nephrolithotomy performed through a sheath too small to accommodate a conventional rigid nephroscope [28]. In a prospective study, Kukreja et al. compared the efficacy and morbidity of reducing the tract size from the standard 24–16.5 Fr for stones measuring 16 to 30 mm. Procedure time, fluoroscopy time, blood loss, pain score, exit strategy, stone clearance status, and complications were evaluated as crucial factors. Miniperc was as effective as conventional PCNL in terms of stone clearance rates. There was no significant difference between the two groups regarding the duration of the procedure or fluoroscopy [29]. In another study, Wishahi et al. found that Mini-PCNL is significantly superior regarding hemoglobin decrease, length of hospital stay, analgesic requirement, and postoperative pain. However, they did not observe a statistically significant difference between Mini PCNL and standard PCNL X-ray exposure times [30]. It is suggested that replacing each dilator in the conventional SAD method alleviates the tamponade effect on the kidney parenchyma and may result in more blood loss during surgery, which is considered a disadvantage of the SAD method [24]. Publications show that dilatation with balloon dilators provides a lower hemoglobin drop than Amplatz and metal telescopic dilators [12]. In contrast, in the Clinical Research Office of the Endourological Society Percutaneous Nephrolithotomy Global Study, BD was associated with more bleeding and transfusion than serial dilatation [31]. In another study, Gonen et al. showed no significant difference between Amplatz dilation and BD regarding bleeding or transfusion rates. They stated that both methods resulted in similar parenchymal damage by expanding the tissue rather than tearing it [32]. However, a meta-analysis showed that OSD significantly reduced the hemoglobin drop compared to serial tract dilation [33]. The current study observed that the hemoglobin drop was statistically significantly lower in the patients in the 3SD group compared to those in the SAD group. However, the two groups had no statistically significant difference regarding the blood transfusion requirement.

Another critical point to consider is that in cases where accurate access from the kidney to the bladder cannot be achieved by the guide wire and is curved in the calyx, multiple dilator inlets and outlets increase the possibility of the guide wire slipping from the access route to the out of the body which causes re-access. Reducing the number of dilators will also reduce this risk. In a study comparing patients who underwent BD and SAD, the loss of passage due to the guide wire coming out of the access tract and the need for re-access were seen only in the SAD group [34]. In our study, four patients in the SAD group required re-access due to the displacement of the guidewire, but this was not observed in any of the cases in the 3SD group.

Open nephrolithotomy may lead to retroperitoneal and perinephric scars around the kidney, adversely affecting the needle’s entry and preventing proper canal expansion, leading to a surgical failure. However, studies have also suggested that it would be better to create the access site away from the previous scar tissue in single-step dilatation and that increasing the number of dilators or using rigid metal dilators may be preferable in cases where dilation over scar tissue is required [24, 35]. In the current study, both 3SD and SAD were successful in patients with a history of open surgery, and there was no need to increase the number of dilators due to the inability to pass the fascia in the 3SD group. We found no significant difference between the two groups regarding tract dilatation success or complications among patients with a previous history of open nephrolithotomy.

Finally, the surgery cost is the most crucial issue, especially in developing countries. Although BD during dilation has certain advantages, such as reducing complication rates related to radiation exposure, this method can be limited due to its high cost. Penbegul et al. designed and used a single 30-F dilator, a 30-F sheath, and an 8-F polyurethane dilator, a method they called the “economic one-shot PCNL set (eco set)” for patients undergoing OSD. They stated that these Amplatz dilator sets could be designed in different numbers according to the surgeon’s needs and that the sets planned this way could reduce the cost of PCNL surgery [36]. In our study, we only used three Amplatz dilators for the patients in the 3SD group. Therefore, similar to Penbegül et al., we consider that dilator sets can be manufactured in different types according to the habits or preferences of each surgeon for operations with lapsed dilatation, which can significantly contribute to the cost-effectiveness of the procedure.

This study showed that the 3SD method could be safely used in PCNL to shorten the time of surgery and fluoroscopy without compromising success or increasing complication rates. Despite our study’s strengths, we acknowledge that it also has several limitations. First, it was conducted in a single center with limited patients, and retrospectively. Second, 3SD was only compared to the conventional SAD, with no other groups being formed in which dilation was performed with OSD or with different numbers of dilators. Multicenter randomized studies with a more significant number of patients and different dilator numbers will provide more comprehensive data. Despite the limitations mentioned earlier, our results indicate that 3SD is as safe and effective as SAD.

## Conclusion

Three-shot dilation can be used as an intermediate step between sequential dilation and one-shot dilation and is an effective and safe method. To determine the optimal number of dilators in PNL, prospective studies with larger numbers of patients are needed.

## Data Availability

All data generated or analyzed during the study are included in this article. Further enquires can be directed to the corresponding author.

## Abbreviations

PCNL: Percutaneous nephrolithotomy
SAD: Sequential amplatz dilatation
MTD: Metal telescopic dilatation
BD: Balloon dilatation
OSD: One-shot dilatation
3SD: Three-shot dilatation
FTs: Fluoroscopy times
CT: Computed tomography
